# Tight and Scalable Side-Channel Attack Evaluations through Asymptotically Optimal Massey-like Inequalities on Guessing Entropy

**DOI:** 10.3390/e23111538

**Published:** 2021-11-18

**Authors:** Andrei Tănăsescu, Marios O. Choudary, Olivier Rioul, Pantelimon George Popescu

**Affiliations:** 1 Department of Computer Science and Engineering, University Politehnica of Bucharest, Splaiul Independentei 313 (6), 060042 Bucharest, Romania; andrei.tanasescu@mail.ru (A.T.); marios.choudary@upb.ro (M.O.C.); 2 LTCI, Télécom Paris, Institut Polytechnique de Paris, 91120 Palaiseau, France; olivier.rioul@telecom-paris.fr

**Keywords:** guessing entropy, side-channel attacks, shannon entropy, massey inequality

## Abstract

The bounds presented at CHES 2017 based on Massey’s guessing entropy represent the most scalable side-channel security evaluation method to date. In this paper, we present an improvement of this method, by determining the asymptotically optimal Massey-like inequality and then further refining it for finite support distributions. The impact of these results is highlighted for side-channel attack evaluations, demonstrating the improvements over the CHES 2017 bounds.

## 1. Introduction

Side-channel attacks on electronic devices have become a very important threat for our society, as shown by European reports [1,2] as well as several recent publications [3,4]. To deal with such threats it is important that devices used in security–critical applications are well protected. Such protection is typically verified through security certifications.

A typical scenario for certifications on cryptographic algorithms such as AES is the estimation of attack success probability as a function of time or data availability. At the beginning of the last decade, a certification would usually include estimating the time necessary for recovering one secret byte from an AES implementation after running a side-channel attack. As such, security metrics such as Success Rate or empirical Guessing Entropy [5] have become popular in this context. But soon afterwards, the question arose how to estimate the probability of success in recovering the entire key (e.g., 16 key bytes for AES-128), not just one byte. In this situation, directly applying the existing Success Rate or Guessing Entropy metrics would not work, due to the impossibility of dealing with all $2^{128}$ possible values of a 16-byte key. Hence, several algorithmic approaches [6,7,8,9,10,11], were developed to estimate e.g., the Guessing Entropy when dealing with full cryptographic keys such as the 16-byte keys used with AES-128.

However, these methods could not scale to deal with very large cryptographic keys, beyond 128 bytes, such as 8912-bit (1024-byte) RSA keys. To deal with this problem, Choudary and Popescu [12] presented a new approach based on mathematical bounds for Massey’s guessing entropy [13]. Their approach could easily handle very large keys, with even beyond 1024 bytes.

Nevertheless, a suggestion was made by Grosso [14] that the bounds of Choudary and Popescu could not be improved, hence this would provide a limitation of that method.

In this paper, we show that this is not the case, by actually tightening the results of Choudary and Popescu, through the derivation of new relations between Massey’s guessing entropy and Shannon’s entropy. These important mathematical results are then validated through concrete side-channel attack experiments.

In brief, the main contributions of this paper are as follows:

1. We demonstrate that a recent improvement on Massey’s inequality between Massey’ Guessing entropy and Shannon’s entropy (Rioul’s improved inequality) is asymptotically optimal (which is highly relevant to scalability).
2. We provide a new improvement on Massey’s inequality that is even tighter than the above for all finite-size data distributions.
3. We extend and prove the above results when dealing with multiple lists of probabilities (distributions), as is the case when dealing with the results of side-channel attacks on multiple key bytes (proving scalability).
4. We apply our results on concrete side-channel attack datasets to demonstrate the improvements of the methods from this paper over the state of the art.

## 2. Preliminaries

The guessing entropy associated with a (positive descending) probability distribution $p=\left(p_{1},\;p_{2},\;\ldots ,\;p_{n}\right)$ with $p_{1}\geq \cdots \geq p_{n}>0$ is the expected value of the random variable $G\left(p\right)$ given by $P\left[G\left(p\right)=i\right]=p_{i}$ (i=1,…,n), i.e., $E\left[G\left(p\right)\right]=\sum_{i=1}^{n}ip_{i}$. It corresponds to the minimal average number of binary questions required to guess the value of a random variable distributed according to p [13]. J. Massey has provided a well-known relation between guessing entropy and the Shannon entropy (In this formula, as well as the remaining of the paper log() denotes logarithm to base 2). $H\left(p\right)=-\sum_{i=1}^{n}p_{i}\log p_{i}$, which reads [13]:

(1) $$E\left[G\left(p\right)\right]\geq 2^{H\left(p\right)-2}+1,$$

when $H\left(p\right)\geq 2$ bits.

Massey’s inequality has been recently improved in various ways, yet all known refinements share the same shape. For instance, in an ISIT paper, Popescu and Choudary [15] proved

$$\begin{array}{c} E\left[G\left(p\right)\right]\geq 2^{H\left(p\right)+2p_{n}-2}+1-p_{n}\geq 2^{H\left(p\right)+p_{n}-2}+1-\frac{1}{2}p_{n}\geq 2^{H\left(p\right)-2}+1, \end{array}$$

subject to the same condition $H\left(p\right)\geq 2$ bits as in the Massey inequality. Meanwhile, Rioul’s inequality [16], published in a CHES paper [17] states that for all values of $H\left(p\right)\geq 0$,

(2) $$E\left[G\left(p\right)\right]>\frac{1}{e}2^{H\left(p\right)},$$

which refines Massey’s inequality when $H\left(p\right)\geq \log\frac{e}{1-e/4}\approx 3$. Finally, in an Entropy paper, Tanasescu and Popescu [18] found that under the same condition as in Massey’s inequality (here h(α) is the binary Shannon entropy),

$$\begin{array}{cc} E\left[G\left(p\right)\right]\geq & \underset{\alpha \in \left[0,1/2\right]}{\sup}2^{H\left(p\right)+\frac{h\left(\alpha \right)}{1-\alpha }p_{n}-2}+1-\frac{\alpha }{1-\alpha }p_{n}\geq 2^{H\left(p\right)+2p_{n}-2}+1-p_{n}>2^{H\left(p\right)-2}+1. \end{array}$$

The authors of [18] hinted that a similar refinement can be found for inequality (2).

In the following section, we present one such refinement, by first optimising exponential relations between the guessing and Shannon entropies, i.e., lower bounds of the form $E\left[G\left(p\right)\right]\geq a\cdot b^{H\left(p\right)}+c$ valid when the Shannon entropy lies above a given threshold. We thus arrive at an improved Rioul’s inequality [19] by an additive constant of 1/2, which is asymptotically optimal among other global lower bounds depending only on the Shannon entropy as $H\left(p\right)\to \infty$ (such condition reflects variables that can take a very large number of values, e.g., when dealing with very large cryptographic keys in our examples). Then, using the techniques of [15,18] we further refine this inequality for finite support distributions allowing us to increase the multiplicative constant depending on the smallest probability $p_{n}$. Finally, then, we apply our results in the context of side-channel attacks, where guessing entropy is a key metric [20,21,22], showing that our results provide an improvement (tighter bounds) over the method of Choudary and Popescu [12], which is known as the most scalable full-key security evaluation method to date.

## 3. The Asymptotically Optimal Massey-like Inequality

Considering the increasingly large key space of cryptographic systems, in this section we seek the best Massey-like inequality $E\left[G\left(p\right)\right]\geq a\cdot b^{H\left(p\right)}+c$, in the sense that it is optimal for arbitrarily large entropy, i.e., when $H\left(p\right)\to \infty$, as can be obtained for infinite support (infinitely large probability lists). Then, we show that this asymptotically optimal bound also holds for all possible distributions.

Recently, [19] proposed an improved version of Rioul’s inequality

(3) $$E\left[G\left(p\right)\right]\geq \frac{1}{e}2^{H\left(p\right)}+\frac{1}{2}.$$

Now we show that as $H\left(p\right)\to \infty$ this inequality is in fact the optimal Massey-like inequality.

**Theorem** **1.** *The Massey-like inequality (3) is asymptotically optimal.*

**Proof.**  Following Massey’s approach [13], the best lower bound on guessing entropy based on Shannon entropy is sharp for geometric distributions, i.e., for any guessing entropy value $E\left[G\left(p\right)\right]=\mu$, the maximal Shannon entropy is obtained for the geometric distribution with mean μ, $p_{i}=\frac{1}{\mu }{\left(1-\frac{1}{\mu }\right)}^{i-1}$,

$$H\left(p\right)\leq \log\left(\mu -1\right)-\mu \log\left(1-1/\mu \right),$$

as found by Massey [13]. Moreover, in practical applications where p has finite length, this inequality is actually strict, but the upper bound can be approached as closely as desired if the list of probabilities is long enough.

We seek bounds of the form $E\left[G\left(p\right)\right]\geq a\cdot b^{H\left(p\right)}+c$, i.e., $H\left(p\right)\leq \log_{b}\frac{\mu -c}{a}$. In order for this to be valid for all μ, we should necessarily have

$$\log_{b}\frac{\mu -c}{a}\geq \log\left(\mu -1\right)-\mu \log\left(1-1/\mu \right).$$

In particular, as μ→∞, the expression on the left has asymptotic

$$\log_{b}\frac{\mu -c}{a}=\log_{b}\mu -\log_{b}a-\frac{c\log_{b}e}{\mu }+o\left(1/\mu \right),$$

while the expression on the right has asymptotic

$$\log\left(\mu -1\right)-\mu \log\left(1-1/\mu \right)=\log\mu +\log e-\frac{\log e}{2\mu }+o\left(1/\mu \right).$$

As a consequence, we necessarily have $\log_{b}\mu \geq \log\mu$, i.e., logb≤1 or b≤2, so that the optimal (maximum) value of *b* is b=2. Next, we should have −loga≥loge, i.e., a≤1/e, so that the optimal (maximum) value of *a* is 1/e. Finally, we should have $-c\log e\geq -\left(\log e\right)/2$, i.e., c≤1/2, so that the optimal (maximum) value of *c* is c=1/2.

The asymptotically optimal bound then writes

(4) $$\log\left(\mu -1/2\right)+\log e\geq \log\left(\mu -1\right)-\mu \log\left(1-1/\mu \right)$$

which readily gives (3) when μ or H(p) tend to infinity.

A simple proof of (3) for all values of H(p)>0 can be found in [19]. □

We conclude this section by remarking that the optimal Massey-like inequality in Theorem 1 is very general, as it also holds for small support corresponding to a few bytes, and even for very small entropy, $H\left(p\right)\to 0$, improving on the original Massey inequality which holds just when $H\left(p\right)\geq 2$.

## 4. Refinement for Finite Support Distributions

In this section, we show a new relation between the Shannon and guessing entropy, dependent on the minimal probability of a given distribution, further refining Rioul’s improved inequality (3).

We begin with a direct improvement of Theorem 1 following the technique [15,18] used to improve the Massey inequality. To this end, we make use of the binary Shannon entropy, $h\left(\alpha \right)=-\alpha \log\alpha -\left(1-\alpha \right)\log\left(1-\alpha \right)$ for 0≤α≤1.

**Lemma** **1.** *For any positive descending probability distribution $p\in R^{n}$ such that $H\left(p\right)\geq 1$ bit, we have*

$$\begin{array}{cc} E\left[G\left(p\right)\right]\geq & \underset{\alpha \in \left[0,1/2\right]}{\sup}\frac{1}{e}2^{H\left(p\right)+p_{n}h\left(\alpha \right)}-\alpha p_{n}+\frac{1}{2}\geq \frac{1}{e}2^{H\left(p\right)+p_{n}}-\frac{1}{2}p_{n}+\frac{1}{2}\geq \frac{1}{e}2^{H\left(p\right)}+\frac{1}{2}. \end{array}$$

**Proof.**  Consider a positive decreasing distribution $p=\left(p_{1},\;p_{2},\;\ldots ,\;p_{n}\right)$ with $H\left(p\right)\geq 2$. Following the approach in [15] we construct the new probability distribution $q=\left(p_{1},\;p_{2},\;\ldots ,\;p_{n-1},\;\left(1-\alpha \right)p_{n},\;\alpha p_{n}\right)$, which is decreasing and strictly positive if and only if $\alpha \in \left(0,\;1/2\right]$. From the grouping property of entropy, $H\left(q\right)=H\left(p\right)+p_{n}h\left(\alpha \right)$, and moreover $E\left[G\left(q\right)\right]=E\left[G\left(p\right)\right]+\alpha p_{n}$. Then

(5) $$\begin{array}{cc} E\left[G\left(p\right)\right]= & E\left[G\left(q\right)\right]-\alpha p_{n}\geq \frac{1}{e}2^{H\left(q\right)}-\alpha p_{n}+\frac{1}{2}\\ = & \frac{1}{e}2^{H\left(p\right)+p_{n}h\left(\alpha \right)}-\alpha p_{n}+\frac{1}{2}. \end{array}$$

The first desired inequality follows taking the supremum over α in Equation (5), the second by substituting α=1/2. To justify the third, we use $2^{x}>1+x\ln2$ for $x=p_{n}$ obtaining

$$\begin{array}{cc} \frac{1}{e}2^{H\left(p\right)+p_{n}}-\frac{1}{2}p_{n}\geq & \frac{1}{e}2^{H\left(p\right)}\left(1+p_{n}\ln\;2\right)-\frac{1}{2}p_{n}=\frac{1}{e}2^{H\left(p\right)}+\left(\frac{2^{H\left(p\right)}\ln2}{e}-\frac{1}{2}\right)p_{n}, \end{array}$$

where $p_{n}$’s coefficient is positive whenever $H\left(p\right)\geq 1\geq \log\frac{e}{2\ln2}$. This ends the proof. □

We can further refine this lemma using the generalization techniques of [15,18] as follows.

**Theorem** **2.** *For any positive descending probability distributions $p\in R^{n}$ such that $H\left(p\right)\geq 1$, we have*

$$\begin{array}{cc} E\left[G\left(p\right)\right]\geq & \underset{\alpha \in \left[0,1/2\right]}{\sup}\frac{1}{e}2^{H\left(p\right)+\frac{h\left(\alpha \right)}{1-\alpha }p_{n}}+\frac{1}{2}-\frac{\alpha }{1-\alpha }p_{n}\geq \frac{1}{e}2^{H\left(p\right)+\frac{1}{2}p_{n}}+\frac{1}{2}-p_{n}\geq \frac{1}{e}2^{H\left(p\right)}+\frac{1}{2}. \end{array}$$

**Proof.**  Given the initial decreasing p, we construct a sequence of probability distributions $\left\{Q_{k}\right\}$, recursively defined using the procedure in the previous proof.

We begin by fixing an arbitrary parameter $\alpha \in \left[0,1/2\right]$ as above. Denoting by $Q_{k,i}$ the *i*^th^ component of the sequence $Q_{k}$, we define the terms of the list $\left\{Q_{k}\right\}$ as follows. We let the support of the first term coincide with p, i.e., $Q_{0}=\left(p_{0},\;p_{1},\;\ldots ,\;p_{n},\;0,\;0,\;\ldots ,\;0,\;\ldots \right)$, and we define the other terms by recurrence:

$$\begin{array}{cc} Q_{k+1}= & \left(Q_{k,0},\;Q_{k,1},\;\ldots ,\;Q_{k,n+k-1},\;\right.\\ \; & \left.\left(1-\alpha \right)Q_{k,n+k},\;\alpha Q_{k,n+k},\;0,\;0,\;\ldots ,\;0,\;\ldots \right). \end{array}$$

and at each step of the construction, we have the inequality

$$\begin{array}{cc} E\left[G\left(Q_{k}\right)\right]= & E\left[G\left(Q_{k+1}\right)\right]-\alpha Q_{k,n+k}\geq \frac{2^{H\left(Q_{k+1}\right)}}{e}-\alpha Q_{k,n+k}>\frac{2^{H\left(Q_{k}\right)}}{e}+\frac{1}{2}. \end{array}$$

After the first *k* steps of the construction we find

$$\begin{array}{cc} E\left[G\left(p\right)\right]= & E\left[G\left(Q_{k}\right)\right]-p_{n}\alpha \frac{1-\alpha^{k}}{1-\alpha }\\ = & E\left[G\left(Q_{k}\right)\right]+\sum_{j=0}^{k-1}\left(E\left[G\left(Q_{j}\right)\right]-E\left[G\left(Q_{j+1}\right)\right]\right)\\ \geq & \frac{1}{2}2^{H\left(Q_{k}\right)}+\frac{1}{2}+\sum_{j=0}^{k-1}\left(E\left[G\left(Q_{j}\right)\right]-E\left[G\left(Q_{j+1}\right)\right]\right)\\ > & \frac{1}{e}2^{H\left(Q_{k-1}\right)}+\frac{1}{2}+\sum_{j=0}^{k-2}\left(E\left[G\left(Q_{j}\right)\right]-E\left[G\left(Q_{j+1}\right)\right]\right)\\ > & \cdots >\frac{1}{e}2^{H\left(Q_{0}\right)}+\frac{1}{2}=\frac{1}{e}2^{H\left(p\right)}+\frac{1}{2}, \end{array}$$

where the tightest of the enumerated bounds is

$$\begin{array}{cc} E\left[G\left(p\right)\right]\geq & \frac{1}{e}2^{H\left(Q_{k}\right)}+\frac{1}{2}+\sum_{j=0}^{k-1}\left(E\left[G\left(Q_{j}\right)\right]-E\left[G\left(Q_{j+1}\right)\right]\right)=\frac{1}{e}2^{H\left(p\right)+p_{n}h\left(\alpha \right)\frac{1-\alpha^{k}}{1-\alpha }}+\frac{1}{2}-p_{n}\alpha \frac{1-\alpha^{k}}{1-\alpha }, \end{array}$$

which as we have shown increases with *k* up to the limit

$$\begin{array}{c} E\left[G\left(p\right)\right]\geq \frac{1}{e}2^{H\left(p\right)+p_{n}\frac{h\left(\alpha \right)}{1-\alpha }}+\frac{1}{2}-p_{n}\frac{\alpha }{1-\alpha } \end{array}$$

valid for any $\alpha \in \left[0,1/2\right]$. The first desired inequality now follows taking *supremum* over the last equation, the second by substituting α=1/2 and the third by noting that all bounds in the sequence are greater than the last one $\frac{1}{e}2^{H\left(p\right)}+\frac{1}{2}$. □

We conclude this section by remarking that Theorem 2 provides a very scalable result as both the Shannon entropy of a joint probability distribution and its minimal entry are very easy to compute, as we will show in the following section.

## 5. Scalability of Bounds

For side-channel attack evaluations on full cryptographic keys (e.g., 16-byte AES keys or 1024-byte RSA keys), we need to combine the attack results on each key byte to derive a security metric that estimates as well as possible the difficulty of recovering the entire key. For example, given the lists of probabilities $p^{1}=\left\{p_{1}^{1},p_{2}^{1},\ldots ,p_{256}^{1}\right\}$, $p^{2}=\left\{p_{1}^{2},p_{2}^{2},\ldots ,p_{256}^{2}\right\}$, …, $p^{16}=\left\{p_{1}^{16},p_{2}^{16},\ldots ,p_{256}^{16}\right\}$ obtained after applying a side-channel attack such as the Template Attack [21,23] for the 16 key bytes of AES, we need security metrics that can use this information efficiently.

In this context, Choudary and Popescu [12] have provided the following bounds (${\mathrm{LB}}_{\mathrm{GM}}$ and ${\mathrm{UB}}_{\mathrm{GM}}$ for full key):

(6) $$\begin{array}{cc} \frac{1}{1+\ln n^{N_{s}}}\prod_{i=1}^{N_{s}}{\left[\sum_{k=1}^{n}\sqrt{p_{k}^{i}}\right]}^{2}\; & \leq E{\left[G\left(p\right)\right]}^{f}\leq \frac{1}{2}\prod_{i=1}^{N_{s}}{\left[\sum_{k=1}^{n}\sqrt{p_{k}^{i}}\right]}^{2}+\frac{1}{2}, \end{array}$$

where $N_{s}$ is the number of bytes (e.g., $N_{s}=16$ for AES-128), *n* represents the number of values per byte (list) and $E{\left[G\left(p\right)\right]}^{f}$ represents the guessing entropy for the full-key (which cannot be computed for large $N_{s}$, e.g., $N_{s}>=10$).

Below we show how to extend the bounds from this paper to apply them in the full-key context.

**Theorem** **3.** *For any full list of probabilities p we have*

$$E{\left[G\left(p\right)\right]}^{f}\geq \frac{1}{e}2^{\sum_{k=1}^{N_{s}}H\left(p^{k}\right)}+\frac{1}{2}.$$

**Theorem** **4.** *For any positive descending probability vectors ${\left\{p^{k}\right\}}_{k=1}^{N_{s}}\subseteq R^{n}$ such that $H{\left(p\right)}^{f}\geq 1$, we have*

$$\begin{array}{cc} \; & E{\left[G\left(p\right)\right]}^{f}\\ \geq & \underset{\alpha \in \left[0,1/2\right]}{\sup}\frac{1}{e}2^{\left(\sum_{k=1}^{N_{s}}H\left(p^{k}\right)\right)+\frac{h\left(\alpha \right)}{1-\alpha }\prod_{k=1}^{N_{s}}p_{n}^{k}}+\frac{1}{2}-\frac{\alpha }{1-\alpha }\prod_{k=1}^{N_{s}}p_{n}^{k}\\ \geq & \frac{1}{e}2^{\left(\sum_{k=1}^{N_{s}}H\left(p^{k}\right)\right)+\frac{1}{2}\prod_{k=1}^{N_{s}}p_{n}^{k}}+\frac{1}{2}-\prod_{k=1}^{N_{s}}p_{n}^{k}\geq \frac{1}{e}2^{\sum_{k=1}^{N_{s}}H\left(p^{k}\right)}+\frac{1}{2}. \end{array}$$

**Proofs.**  Both results follow immediately from Theorems 1 and 2 considering the additivity of the Shannon entropy,

$$H{\left(p\right)}^{f}=H\left(\underset{k=1}{\overset{N_{s}}{⊗}}p^{k}\right)=\underset{k=1}{\sum^{N_{s}}}H\left(p^{k}\right),$$

and the fact that the minimal entry in the full list of probabilities p is the product of the individual minima $p_{n}^{k}$. □

In conclusion, we presented the optimal Massey-like inequality for the full-key context in the form of Theorem 3 as well as an improvement of it, Theorem 4, showing that our results are indeed highly scalable.

For practical purposes, given that limited representation of numbers may lead to zero values in large lists of probabilities, in our experiments we consider as $p_{n}$ the least non-zero probability, i.e., $p_{n}>0$, which leads to accurate results for the bounds presented in this paper.

As a final remark, we note here that our new improvement which most completely manifests in the form of Theorem 2 is tight whenever the smallest non-zero probability is significant, such as uniform distributions or geometric distributions with truncated tail, but can also be beneficial for other classes of distributions, such as those encountered in side-channel attack evaluation, as will be discussed in the next section.

## 6. Evaluation on Side-Channel Attack Data

As mentioned in the introduction, in many security-critical applications, such as banking or physical access control, it is imperative to use hardware that is security certified. In order to obtain a security certification such as those offered by Common Criteria [24] it is also typically necessary to prove that a device is resilient to side-channel attacks and this is generally done by showing that the guessing entropy or some other security metric is within certain thresholds. In this context, the bounds from this paper represent a very useful tool for a security evaluator, as they allow improving the tightness of the CHES2017 bounds, which are considered to be the most scalable tool to date for evaluating the security of cryptographic algorithms, allowing security estimation when dealing with very large keys. Hence, in this section we demonstrate the relevance of the results from this paper, by comparing the scalable versions of our bounds (see previous sections) against the bounds from CHES 2017.

### 6.1. Evaluation Data

To easily compare the bounds from this paper against the CHES 2017 method of Choudary and Popescu [12], we used the same datasets as in the CHES 2017 paper: (*a*) a simulated dataset (MATLAB generated power consumption from the execution of the AES S-box) and (*b*) a real dataset (power traces from the execution of AES in the AES hardware engine of an AVR XMEGA microcontroller).

In both experiments, the AES encryption algorithm is used with 128-bit (16-byte) keys. The AES state is composed of 16 bytes, which are processed sequentially within certain operations such as the Sub Bytes (S-Box) operation, which is the typical target of side-channel attacks, including ours.

The steps for our experiments are as follows:

1. For each dataset (power traces), we run a Template Attack [23] using the set of power traces to determine the most likely value of each of the 16 bytes of the AES key. The result of this attack is a list of probabilities $p^{k}=\left\{p_{1},p_{2},\ldots ,p_{256}\right\}$ for each of the 16 bytes of the AES key ($K=[k_{1}k_{2}\ldots k_{16}]$).
2. Using the lists of probabilities $p^{1},p^{2},\ldots ,p^{16}$, we compute the bounds (those from this paper as well as those from CHES 2017) first for each byte individually and then for attacks on two or more key bytes. Please note that a direct computation of the guessing entropy through the computation of the cross-product of several lists of probabilities (e.g., for more than 8 key bytes) is not feasible as we would have to process lists of more than $2^{64}$ elements. Instead, the bounds from this paper (as well as those from CHES 2017) use directly and very efficiently the lists of probabilities for each key byte, without performing the cross-product, to derive security metrics for attacks on many target bytes.

In the following, we present the results of our evaluations for three interesting cases: (1) application of the bounds on single lists of probabilities—this is equivalent to attacking a single key byte in side-channel attack evaluations; (2) application of the bounds when targeting two bytes—this is interesting to test the scalability of the bounds; (3) application of the bounds when attacking 16 bytes—this represents a complete attack on the full AES key and hence is a representative scenario of a full-fledged security evaluation, where scalability and tightness are very important.

### 6.2. Evaluation on a Single Byte

**Figure 1:**
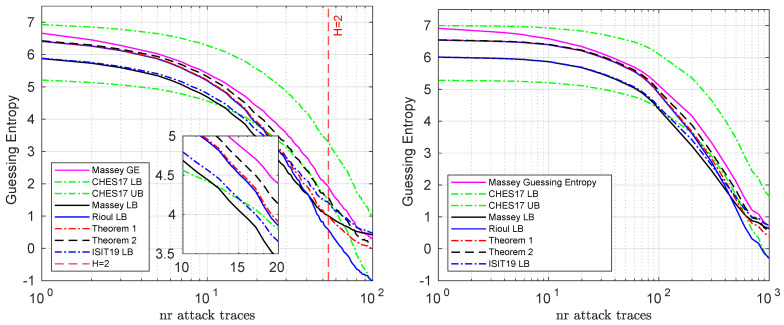
Bounds for the simulated (**left**) and real (**right**) datasets, when targeting a single subkey byte. These are averaged results over 100 experiments.

We show the bounds for a single key byte on the simulated and real datasets in Figure 1. Here we can see that while the CHES lower bound is tighter when the guessing entropy is low (below 4 bits), in the other (most) cases Rioul’s lower bound is better. Furthermore, we can see that Theorem 1 provides a better (tighter) lower bound than Rioul’s lower bound and Theorem 2 in turn provides an even better lower bound than Theorem 1.

An interesting artifact appears when the guessing entropy decreases below two bits (log(G(p))=1), where the Massey inequality (and the ones in ISIT 2019 [15]) does not necessarily hold (considering for example geometric distributions with $p_{1}\geq 1/2$). In this case, most bounds do not hold anymore. Meanwhile, bounds based on Rioul’s inequality (Rioul LB, Theorem 1, Theorem 2) all continue to hold in this regime, owing to the fact that it does not impose preconditions on the minimal value of H(p).

For reference, in Figure 1 we included all present refinements of Massey’s inequality. However, for clarity, in the following sections we will only compare our bounds with CHES 2017.

### 6.3. Evaluation on Two Bytes

**Figure 2:**
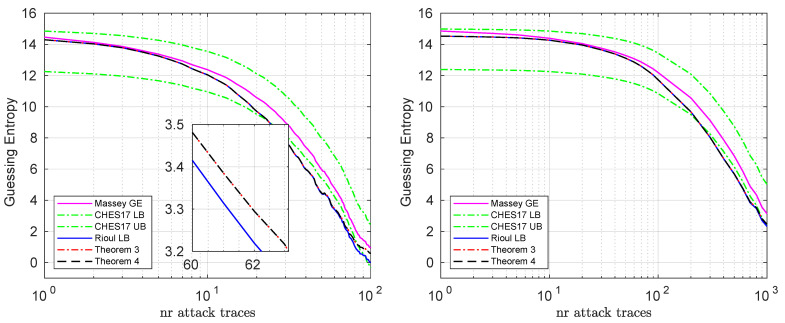
Bounds for the simulated (**left**) and real (**right**) datasets, when targeting two subkey bytes. These are averaged results over 100 experiments.

We show the bounds when targeting two key bytes on the simulated and real datasets in Figure 2. Here we see again that Rioul’s bound is tight when the guessing entropy is higher, but then the CHES lower bound becomes tighter, as the guessing entropy decreases. We can also confirm here that our theorems provide a better (tighter) lower bound than Rioul’s lower bound.

### 6.4. Evaluation on All 16 Bytes

**Figure 3:**
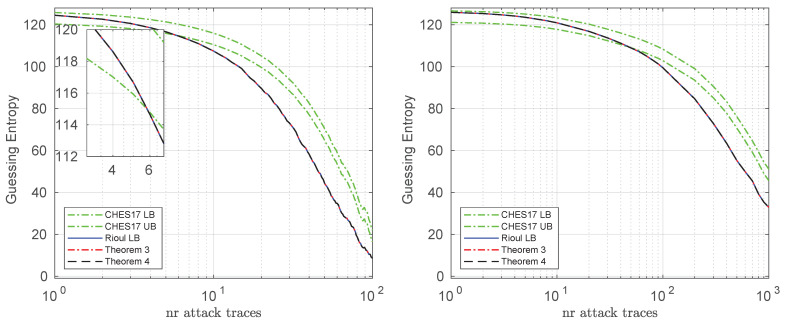
Bounds for the simulated (**left**) and real (**right**) datasets, when targeting all the 16 AES key bytes. These are averaged results over 100 experiments.

Finally, we show the bounds when targeting all the 16 bytes of the full AES key on the simulated and real datasets in Figure 3. We did not plot the actual value of the guessing entropy in this case, because it is not possible to compute it: it would require the iteration over (and sorting of) a list of $2^{128}$ elements. Hence, in this case the computationally efficient bounds compared in this paper become very valuable. From the figure we see again that when the guessing entropy is very high (e.g., above 120 bits), all the lower bounds presented in this paper are tighter than the CHES 2017 lower bound (Theorems 3 and 4 provide numerically similar results to Rioul’s lower bound), hence tightening the security evaluation results for larger values of the guessing entropy. This allows for an overall improved method than that of CHES 2017 (e.g., by taking the maximum between the CHES 2017 lower bound and Theorems 3/4).

## 7. Conclusions

In this paper, we have improved the security evaluation metric of Choudary and Popescu from CHES 2017, which is considered the most scalable method to date, by tightening Massey’s inequality even further. First, we have demonstrated that the improved Rioul’s inequality is asymptotically optimal and showed how to scale this method for use in full-key evaluation methods. Then, using the techniques of [15,18], we further refined this inequality for finite support distributions allowing us to increase the multiplicative constant depending on the smallest probability $p_{n}$. We compared all our results to those of Choudary and Popescu from CHES 2017 using their datasets, demonstrating the usefulness of the improvements from this paper.

For future work we are very interested in further results based on other (additive) entropies, such as Rényi entropies where other guessing bounds are already investigated [19] past their original use in moment inequalities [25,26,27] and other derived problems such as guessing with limited (or no) memory [28].

## Data Availability

Not applicable.
